# Risk Factors for Slow Viral Decline in COVID-19 Patients during the 2022 Omicron Wave

**DOI:** 10.3390/v14081714

**Published:** 2022-08-04

**Authors:** Xin Li, Anthony Raymond Tam, Wing-Ming Chu, Wan-Mui Chan, Jonathan Daniel Ip, Allen Wing-Ho Chu, Syed Muhammad Umer Abdullah, Cyril Chik-Yan Yip, Kwok-Hung Chan, Samson Sai-Yin Wong, Vincent Chi-Chung Cheng, Kwok-Yung Yuen, Ivan Fan-Ngai Hung, Kelvin Kai-Wang To

**Affiliations:** 1 State Key Laboratory for Emerging Infectious Diseases, Carol Yu Centre for Infection, Department of Microbiology, School of Clinical Medicine, Li Ka Shing Faculty of Medicine, The University of Hong Kong, Pokfulam, Hong Kong SAR, China; 2 Department of Microbiology, Queen Mary Hospital, Hong Kong SAR, China; 3 Department of Medicine, Queen Mary Hospital, Hong Kong SAR, China; 4 Infection Control Team, Queen Mary Hospital, Hong Kong West Cluster, Hong Kong SAR, China; 5 Guangzhou Laboratory, Guangzhou 510725, China; 6 Department of Medicine, School of Clinical Medicine, Li Ka Shing Faculty of Medicine, The University of Hong Kong, Pokfulam, Hong Kong SAR, China

**Keywords:** SARS-CoV-2, COVID-19, omicron variant, delta variant, viral shedding, geriatrics

## Abstract

Formulating termination of isolation (de-isolation) policies requires up-to-date knowledge about viral shedding dynamics. However, current de-isolation policies are largely based on viral load data obtained before the emergence of Omicron variant. In this retrospective cohort study involving adult patients hospitalised for COVID-19 between January and February 2022, we sought to determine SARS-CoV-2 viral shedding kinetics and to investigate the risk factors associated with slow viral decline during the 2022 Omicron wave. A total of 104 patients were included. The viral load was highest (Ct value was lowest) on days 1 post-symptom-onset (PSO) and gradually declined. Older age, hypertension, hyperlipidaemia and chronic kidney disease were associated with slow viral decline in the univariate analysis on both day 7 and day 10 PSO, while incomplete or no vaccination was associated with slow viral decline on day 7 PSO only. However, older age was the only risk factor that remained statistically significant in the multivariate analysis. In conclusion, older age is an independent risk factor associated with slow viral decline in this study conducted during the Omicron-dominant 2022 COVID-19 wave. Transmission-based precaution guidelines should take age into consideration when determining the timing of de-isolation.

## 1. Introduction

Severe acute respiratory syndrome coronavirus 2 (SARS-CoV-2) is one of the most contagious respiratory viruses [1]. The Omicron variant, first emerged in South Africa in November 2021, is particularly transmissible, with an estimated growth rate 3.5 times faster than that of Delta variant [2]. In particular, BA.2 sub-lineage is more transmissible than BA.1 [3,4]. In Hong Kong, the Omicron variant has caused the fifth wave of COVID-19, and the sub-lineage BA.2.2 is the predominant lineage since late January 2022 [3,5].

Home isolation is an important public health measure to reduce community spread, while cohort or single-room isolation can prevent nosocomial transmission within healthcare facilities. Symptom-based approach is currently used in most places for the determination of the duration of isolation. The recommended duration of home isolation in many countries is often 5–7 days [6,7,8,9,10]. In hospital settings, the US Centers for Disease Control and Prevention and the UK Health Security Agency recommend that a COVID-19 patient should be isolated in a single-person room for at least ten days, until fever subsides and symptoms are improving, except for immunocompromised patients for whom a test-based strategy is required for ending isolation [11,12].

Although symptom-based isolation policy is convenient, it does not take into account other factors which have been shown to affect the duration of high viral load shedding [13,14,15,16,17,18]. Furthermore, since current isolation policies are mainly based on viral load dynamics data collected before the emergence of the Omicron variant [11], these may not be applicable to patients infected by the Omicron variant. Moreover, previous studies on each host factor were often analysed individually without performing multivariate analysis to exclude potential confounding factors. Here, we address these deficiencies by assessing the viral load kinetics among COVID-19 patients during the 2022 Omicron wave and used univariate and multivariate analyses to determine risk factors affecting viral decline.

## 2. Materials and Methods

### 2.1. Patients

This is a retrospective cohort study involving adult patients admitted to Queen Mary Hospital for laboratory-confirmed COVID-19 between 20 January 2022 and 25 February 2022. In Hong Kong, the cycle threshold (Ct) value was used as one of the discharge criteria during this period (Table 1). Therefore, even patients who were asymptomatic or had clinically improved were required to be hospitalised until the Ct values met the discharge criteria. In our hospital, saliva was used for monitoring viral load because saliva is a non-invasive type of specimen, and it has been shown to demonstrate less variation in human RNase P Ct value than nasopharyngeal swabs [19].

Patients were included if (i) aged 18 years or above; (ii) at least one saliva specimen tested positive for SARS-CoV-2 by real-time reverse transcription-polymerase chain reaction (RT-PCR), and (iii) at least one saliva specimen was available on or after day 7. Patients were excluded if clinical information was not available or if COVID-19 vaccination status was unknown. The clinical details of each patient, including the demographics, chronic comorbidities, COVID-19 vaccination details, severity of infection and treatment received, were retrieved from the electronic patient record. We defined severe disease as the need for supplemental oxygen. The study was approved by the Institutional Review Board of The University of Hong Kong/Hospital Authority Hong Kong West Cluster (UW 22-052). Since patients were recruited retrospectively and archived specimens were used, written informed consent was waived.

### 2.2. Definitions

Patients were considered to be fully vaccinated if they have received at least 2 doses of COVID-19 vaccines at least 14 days prior to symptom onset for symptomatic patients or the first positive SARS-CoV-2 test for asymptomatic patients. For the purpose of assessing viral kinetics, day 0 was taken as the day of the first positive SARS-CoV-2 test for asymptomatic patients.

A patient was considered to have slow viral decline (SVD) on day 7 PSO if the Ct value was <30 for any specimen collected on or after day 7 PSO (SVD-7). Patients were considered to have rapid viral decline (RVD) on day 7 PSO if they did not fulfil the criteria for SVD. The Ct value cut-off of 30 was chosen to differentiate SVD and RVD because a previous study has shown that transmission was lower for patients with Ct values of >30 than those with Ct < 30 [20]. Furthermore, several studies have shown that specimens with Ct values of >30 are unlikely to yield live virus [21].

We assessed SVD and RVD both on day 7 and day 10 PSO because the de-isolation criteria for hospitalised patients vary from day 7 or 10 according to different guidelines and situations. The criteria for classifying a patient as SVD or RVD for day 10 PSO were similar to those of day 7 PSO, except that (i) all patients with RVD on day 7 PSO were considered to have RVD on day 10 PSO; and (ii) a patient was excluded from day 10 PSO analysis if no specimens were collected on or after day 10 PSO.

### 2.3. Saliva Specimens SARS-CoV-2 RT-PCR and Whole Genome Sequencing

Saliva specimens were collected as described previously [22]. Patients were instructed to submit around 1 mL of saliva directly into a sterile bottle. Upon arrival at the laboratory, phosphate buffered saline was added to the specimen to top up the volume to 2 mL. During the study period, all saliva specimens submitted to our laboratory for urgent SARS-CoV-2 RT-PCR were tested by the by the Xpert Xpress SARS-CoV-2 assay (Cepheid, Sunnyvale, CA, USA) which targeted the E and N genes of SARS-CoV-2, according to manufacturer’s instructions. Briefly, 300 μL of each specimen was directly added into the Xpert cartridge, which was loaded into the GeneXpert XVI system (Cepheid, Sunnyvale, CA, USA). Routine specimens (those not tagged as “urgent”) were tested by a commercial SARS-CoV-2 real-time RT-PCR targeting the E gene (TIB Molbiol, Berlin, Germany). The SARS-CoV-2 lineage was determined by whole genome sequencing using the Oxford Nanopore MinION device (Oxford Nanopore Technologies, Oxford, UK) as described previously [3].

### 2.4. Statistical Analysis

Statistical analysis was performed using IBM SPSS Statistics 28.0.1.0 or PRISM version 9.1.2 (GraphPad Software, San Diego, CA, USA). Categorical variables were compared using Fisher’s exact test and continuous variables compared using Mann–Whitney U test. Multivariable logistic regression models were built using backward stepwise elimination (likelihood ratio) method with a *p* value of <0.1 required for inclusion. A 2-sided *p* value of <0.05 was considered statistically significant. Graphs were created using PRISM.

## 3. Results

### 3.1. Patient Characteristics

During the study period, there were a total of 211 hospitalised adult patients with at least one saliva specimens tested positive for SARS-CoV-2, and 104 fulfilled the inclusion and exclusion criteria and had sufficient specimens available to determine the viral decline on day 7 PSO (Table 2). The median age was 68 years (interquartile range: 47–76), and 44.2% (46/104) were female. Chronic medical illness was present in 70.2% (73/104) of patients, and 49% (51/104) were fully vaccinated. Remdesivir was given to 27.9% (29/104) of patients, respectively, and 2.9% (3/104) required oxygen supplementation. Whole viral genome sequencing showed that 90.4% (94/104) and 9.6% (10/104) were infected with the Omicron and Delta variants, respectively.

**Figure 1:**
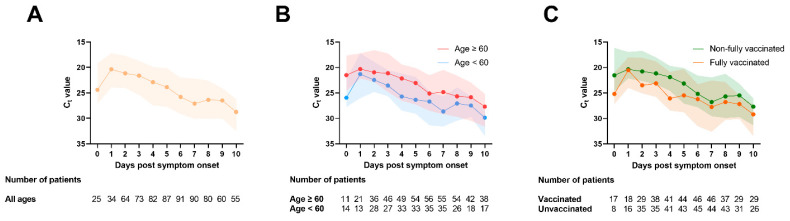
Serial changes of Ct value in saliva specimens. (**A**) All patients; (**B**) according to age; (**C**) according to vaccination status. Each dot represents the median viral load, and the shading represents the interquartile range.

Figure 1 shows the overall trend of SARS-CoV-2 RNA shedding in saliva in the first 10 days PSO. The viral load was highest (Ct value was lowest) on day 1 PSO, and gradually decreased (Figure 1A). There was no difference in the median peak viral loads between older and younger individuals (Figure 1B) and between fully vaccinated and non-fully vaccinated patients (Figure 1C). However, on or after day 2 PSO, the viral load was generally lower in younger adults (<60 years old) than older adults (≥60 years old) and in fully vaccinated than non-fully vaccinated individuals.

### 3.2. Risk Factors Associated with Slow Viral Decline in Saliva

To assess for risk factors associated with prolonged high-level RNA shedding, we classified patients into two groups, SVD and RVD (see methods for the criteria). Univariate analysis showed that older age, presence of chronic comorbidities, hypertension, hyperlipidaemia, and chronic kidney disease were statistically significantly associated with SVD on both day 7 and 10 PSO (Table 2), while connective tissue disease was significantly associated with SVD on day 10 PSO only. The proportion of fully vaccinated patients was statistically significantly lower in the SVD than RVD group, but only reached statistical significance on day 7 PSO (44% (37/84) for SVD vs. 70% (14/20) for RVD; *p* = 0.047). Notably, disease severity (symptomatic or oxygen requirement) and treatment (remdesivir) were not significantly associated with viral decline on either day 7 or 10 PSO.

Since previous studies demonstrated that the neutralising antibody levels were lower in patients who received CoronaVac vaccine compared with those who received BNT162b2 vaccine [23], we performed sub-group analysis for the fully-vaccinated patients. There was no significant difference in the proportion of patients receiving BNT162b2 between the SVD and RVD group on either day 7 (*p* = 0.525) or day 10 PSO (*p* = 0.547).

In order to determine independent host factors associated with slower viral clearance, we performed a multivariate analysis. Only older age remained statistically significantly associated with SVD on both day 7 and day 10 PSO (Day 7: *p* = 0.016; Day 10: *p* = 0.018).

## 4. Discussion

Viral shedding dynamics is a major consideration when formulating policies on de-isolation of COVID-19 patients. Patients with higher viral loads (lower Ct values) are more likely to transmit the virus to others [24]. In this study, older age was the only independent host factor significantly associated with slower viral decline. Aging is associated with immunosenescence, which affects both the innate and the adaptive immune systems [25]. In our previous in vivo study, aged mice showed higher viral loads in the nasal turbinate and in the lung, but weaker interferon and antibody response, than young mice (6–8 weeks old) [26]. Aging is also associated with T cell exhaustion [27], but the role of T cell in viral clearance during SARS-CoV-2 infection remains to be determined [28].

In a large study involving over 10,000 individuals, Levine-Tiefenbrun et al. found that fully vaccinated individuals had lower viral loads than non-vaccinated individuals [17]. Another cohort study of healthcare workers by Jung J et al. found that fully vaccinated individuals had a shorter duration of viable viral shedding [29]. In our current study, although there was a tendency towards lower viral load among fully vaccinated individuals, multivariate analysis revealed that vaccination status was not an independent risk factor associated with SVD. Our results concurred with a recent study by Boucau J et al., which showed no significant difference in the time to PCR conversion or culture conversion between non-vaccinated and vaccinated individuals infected with Delta or Omicron variants [30]. The apparent contradictory findings may be related to the different settings. First, our cohort had a median age of 68 years old, which was much older than those in the studies by Levine-Tiefenbrun et al. (median age, 42 years) and Jung J et al. (median age, 47 years). Hence, we were able to evaluate viral decline in these older adults. Second, our study was conducted during the Omicron wave. The neutralizing antibody titers against the Omicron variant were much lower than those against the ancestral virus due to immune escape [31,32,33]. As a result, vaccine effectiveness seems to be much reduced against the Omicron variant than against previous strains.

We chose a Ct value of 30 as the cut-off for SVD and RVD because the main aim of this study was to provide data for guiding de-isolation policy. In the study by Kim et al., which assessed serial specimens from hospitalised patients, only specimens with a Ct of 28.4 were culturable [22]. Takahashi et al. showed that for Omicron patients, only those with Ct of <30 can be cultured [34].

We did not find any differences between patients with prior CoronaVac and BNT162b2 vaccination. Previous studies showed that the neutralizing antibody titers elicited by BNT162b2 were much higher than by CoronaVac [23]. Therefore, viral clearance in the upper respiratory tract may not correlate with serum neutralizing antibody titers, but it depends on a complex interplay between humoral and cell-mediated immune pathways. Whether the intranasal vaccine, which elicits better mucosal immune response in the airway, can hasten the decline in viral load in the upper respiratory tract remains to be determined [35].

There are several limitations in this study. First, we could not determine the effect of three doses of vaccine as there were only seven patients who had received booster dose vaccine. Multiple studies have demonstrated that a booster dose of vaccine can significantly increase the neutralization titers against the Omicron variant [36], which may accelerate the decline in viral load. Second, none of the patients received the oral antivirals molnupiravir or nirmatrelvir-ritonavir as these were not available in Hong Kong during the study period. Previous studies suggest that viral load at day 5 of treatment decreased by 0.3 log10 copies/mL for molnupiravir and 0.87 log10 copies/mL for nirmatrelvir-ritonavir compared with placebo [37,38]. Further studies should be conducted to evaluate the reduction in viral load for these oral drugs in real-life situations. Third, there were too few severe cases in our cohort to determine whether patients with severe disease require a longer duration of isolation. Similarly, many of the comparisons in the univariate analysis were limited by small sample size, such as immunocompromised state and connective tissue disease, which may limit the power to detect significant association. Fourth, we selected two time points, 7 and 10 days PSO, to assess the viral shedding since most guidelines stipulated an isolation duration of 7 to 10 days. However, both intermittent viral shedding and prolonged detection of viral RNA have been reported in COVID-19 patients [22]. More frequent collection of specimens for extended duration is required to better delineate the temporal profile. Finally, we only included patients with at least one saliva specimen available on or after day 7. Since some patients may have been discharged before day 7 by meeting the serological and Ct value criteria (Table 1), this may cause selection bias in the cohort of patients included in the analysis.

In conclusion, after adjusting for confounding factors, only older age remained to be an independent risk factor associated with SVD. Viral load monitoring by real-time RT-PCR or antigen test may be useful in guiding decision for de-isolation among older adults.

## Figures and Tables

**Table 1:** Evolution of discharge criteria for laboratory-confirmed COVID-19 patients in Hong Kong.

Effective Date	Criteria for Releasing Confirmed COVID-19 Patients from Isolation
27 October 2021 to 1 February 2022	**For symptomatic patients:** (a) Afebrile for >3 days; AND (b) Significant improvement in respiratory symptoms; AND (c) Significant improvement in lung infiltrates in chest imaging; AND (d) With two clinical specimens of the same type (i.e., respiratory or stool) taken at least 24 h apart tested negative by RT-PCR; AND (e) 10 days have passed since the onset of illness. **For patients who did not develop any COVID-19–compatible symptoms all along:** With two clinical specimens of the same type (i.e., respiratory or stool) taken at least 24 h apart tested negative by RT-PCR; AND 10 days after the first positive RT-PCR test for SARS-CoV-2
2 February 2022 to 11 February 2022	**For symptomatic patients** (a) Clinical conditions improve and afebrile; AND (b) Either one of the following criteria: With two clinical specimens of the same type (i.e., respiratory or stool) taken at least 24 h apart tested negative RT-PCR; OR three clinical specimens of the same type taken at least 24 h apart in which RT-PCR test results showed consistent Ct value 33 or above; AND 10 days have passed since the onset of illness; OR With a transition of the test results for SARS-CoV-2 IgG from negative to positive with at least one PCR Ct value 33 or above. **For patients who did not develop any COVID-19–compatible symptoms all along** (a) Either one of the following laboratory criteria: With two clinical specimens of the same type (i.e., respiratory or stool) taken at least 24 h apart tested negative by RT-PCR; OR three clinical specimens of the same type taken at least 24 h apart in which RT-PCR test results showed consistent Ct value 33 or above; AND 10 days after the first positive RT-PCR for SARS-CoV-2; OR Serology test result for SARS-CoV-2 IgG change from negative to positive with at least one PCR Ct value 33 or above.
12 February 2022 to 25 February 2022	Clinical conditions improve and afebrile (for symptomatic patients); AND Either one of the following criteria: (a) Two respiratory specimens (or stool samples if applicable) taken at least 24 h apart in which RT-PCR test results showed consistent Ct value 30 or above; OR (b) With the initial SARS-CoV-2 RBD IgG test result positive while an increasing trend of IgG observed AND at least one negative RAT result AND at least one RT-PCR Ct value 30 or above; OR (c) With a transition from a negative SARS-CoV-2 IgG test result to positive AND at least one RT-PCR Ct value 30 or above.

**Table 2:** Comparison between patients with slow viral decline (SVD) and rapid viral clearance (RVD) on day 7 PSO.

	Day 7 PSO				Day 10 PSO			
	All Patients n = 104	SVD ^a^ n = 84	RVD n = 20	*p* Value	All Patients n = 79	SVD ^b^ n = 45	RVD n = 34	*p* Value
**Demographics**								
Median age in years (interquartile range)	68 (47–76)	69 (53–76)	47 (34–74)	0.033	68 (45–76)	70 (55–80)	53 (36–74)	0.019
Age 60 years or above	66 (63.5)	58 (69)	8 (40)	0.021	48 (60.8)	33 (73.3)	15 (44.1)	0.011
Female sex	58 (55.8)	50 (59.5)	8 (40)	0.137	41 (51.9)	27 (60)	14 (41.2)	0.155
**Chronic comorbidities**								
Presence of chronic comorbidities	73 (70.2)	64 (76.2)	9 (45)	0.012	52 (65.8)	36 (80)	16 (47.1)	0.004
Hypertension	48 (46.2)	44 (52.4)	4 (20)	0.012	34 (43)	25 (55.6)	9 (26.5)	0.012
Hyperlipidaemia	41 (39.4)	38 (45.2)	3 (15)	0.020	28 (35.4)	21 (46.7)	7 (20.6)	0.019
Diabetes mellitus	26 (25)	24 (28.6)	2 (10)	0.148	15 (19)	11 (24.4)	4 (11.8)	0.246
Neurologic/cognitive disease	22 (21.2)	19 (22.6)	3 (15)	0.555	15 (19)	11 (24.4)	4 (11.8)	0.246
Chronic heart disease	16 (15.4)	14 (16.7)	2 (10)	0.731	11 (13.9)	8 (17.8)	3 (8.8)	0.335
Chronic kidney disease	17 (16.3)	17 (20.2)	0 (0)	0.038	10 (12.7)	9 (20)	1 (2.9)	0.037
Immunocompromised state	5 (4.8)	4 (4.8)	1 (5)	1.000	3 (3.8)	1 (2.2)	2 (5.9)	0.574
Chronic liver disease	6 (5.8)	6 (7.1)	0 (0)	0.593	4 (5.1)	3 (6.7)	1 (2.9)	0.630
Connective tissue disease	6 (5.8)	6 (7.1)	0 (0)	0.593	6 (7.6)	6 (13.3)	0 (0)	0.034
Pulmonary disease	8 (7.7)	6 (7.1)	2 (10)	0.648	6 (7.6)	3 (6.7)	3 (8.8)	1.000
**COVID-19 vaccination history**								
Fully vaccinated ^1^	51 (49)	37 (44)	14 (70)	0.047	46 (58.2)	24 (53.3)	22 (64.7)	0.362
BNT162b2 ^2^	51 (100)	25 (67.6)	8 (57.1)	0.525	28 (60.9)	16 (66.7)	12 (54.5)	0.547
Booster dose ^2^	7 (13.7)	6 (16.2)	1 (7.1)	0.657	6 (13)	5 (20.8)	1 (4.5)	0.190
**Lineage**								
Omicron	94 (90.4)	75 (89.3)	19 (95)	0.683	69 (87.3)	38 (84.4)	31 (91.2)	0.502
**Treatment**								
Remdesivir	29 (27.9)	22 (26.2)	7 (35)	0.421	24 (30.4)	15 (33.3)	9 (26.5)	0.623
**Severity of disease**								
Symptomatic	78 (75)	63 (75)	15 (75)	1.000	62 (78.5)	35 (77.8)	27 (79.4)	>0.999
Require O2	3 (2.9)	3 (3.6)	0 (0)	1.000	2 (2.5)	1 (2.2)	1 (2.9)	1.000

^1^ Received at least 2 doses of COVID-19 vaccines 14 days prior to symptom onset or first positive SARS-CoV-2 test. ^2^ Percentage of fully vaccinated individuals. Abbreviations: IQR, interquartile range; RVD, rapid viral decline; SVD, slow viral decline. ^a^—if the Ct value was <30 for any specimen collected on or after day 7 PSO. ^b^—if the Ct value was 30 for any specimen collected on or after day 10 PSO.

## Data Availability

Data are contained within the article. Further enquires can be directed to corresponding author.
